# The use of *Drosophila* to study sleep impairments in stimulant use disorder

**DOI:** 10.3389/fcell.2026.1913395

**Published:** 2026-09-03

**Authors:** Madelyn Miles, Mert Karatas, Adrian Rothenfluh

**Affiliations:** 1 Neuroscience Graduate Program, University of Utah, Salt Lake City, UT, United States; 2 Department of Psychiatry, Huntsman Mental Health Institute, University of Utah, Salt Lake City, UT, United States; 3 Molecular Medicine Program, University of Utah, Salt Lake City, UT, United States; 4 Department of Neurobiology, University of Utah, Salt Lake City, UT, United States; 5 Department of Human Genetics, University of Utah, Salt Lake City, UT, United States

**Keywords:** addiction, dopamine, *Drosophila*, psychostimulants, sleep

## Abstract

Stimulant drugs like cocaine and methamphetamine acutely enhance the release of monoamine neurotransmitters, particularly dopamine, to induce euphoria, motivation, hyperlocomotion, and strong sleep suppression. Chronic stimulant use can lead to the development of stimulant use disorder (SUD), which is characterized by risky, excessive, or uncontrolled drug intake and an inability to cease use. At time of writing, there is no FDA-approved pharmacological treatment for SUD. Though symptoms and comorbidities of SUD vary, studies on abstinent stimulant-users repeatedly identify sleep disorder as a symptom that persists long into abstinence and drives relapse to stimulant use. This review discusses the use of the vinegar fly *Drosophila melanogaster* to identify the neural and genetic pathways mediating this sleep disorder. The fly has a long history of use in neurogenetics research, boasting efficient and low-cost husbandry, an extremely well-studied genome, widely available tools for manipulating neurons and genes with high spatiotemporal specificity, and a connectome cataloguing each neuron and synapse in the fly brain. Additionally, the fly rest state is a face-valid model of sleep in mammals, as it shares key features and is regulated by conserved neurotransmitter systems; flies also respond similarly to stimulant drugs, again mediated by common monoamine signals. As such, flies represent an excellent and under-utilized invertebrate model for studying sleep deficits in stimulant abstinence. This review describes methods available for studying sleep and stimulant use in *Drosophila*, highlighting key findings in the existing literature relevant to this phenotype while indicating where the literature is lacking. Identification of the genetic and neural pathways mediating stimulant-induced sleep disorder in flies will shed light on the critical role of dopamine systems in sleep regulation, facilitating the development of more efficacious treatments for SUD.

## 1 Introduction to stimulant use disorder and sleep

### 1.1 Stimulant use disorder

Stimulants are drugs that acutely activate the central nervous system (CNS) to increase alertness and arousal. This drug class includes prescription stimulants such as methylphenidate (Ritalin) and lisdexamfetamine (Vyvanse), as well as illicit stimulants such as methamphetamine, amphetamine, cocaine, and 3,4-methylenedioxymethamphetamine (MDMA, “ecstasy”). Stimulant use disorder (SUD) is defined as a pattern of cocaine, amphetamine-type substance, or other stimulant use leading to clinically significant impairment or distress (Diagnostic and statistical manual of mental disorders: DSM-5, 2013). Clinically, SUD is marked by heavy use despite negative consequences, unsuccessful attempts to cease use, tolerance to drug effects, withdrawal symptoms upon cessation of use, and symptoms that chronically impact mood, cognition, and daily functioning.

SUD represents a substantial and growing public health burden globally. In the U.S., an estimated 1.5% of people aged 12+ (4.3 million) met criteria for past-year CNS stimulant use in 2024 (Substance Abuse and Mental Health Services Administration, 2025). Alarmingly, overdose deaths involving stimulants have markedly increased over the past decade. For example, in 2023, there were 29,449 deaths involving cocaine in the U.S., compared to 4,944 only a decade previously (Garnett, 2024); this sharp increase has also been observed in countries including Brazil, Canada, France, and the United Kingdom (United Nations Office on Drugs and Crime, 2025). Global methamphetamine prevalence is likewise increasing, indicated by a 31% increase in the quantity of methamphetamine seized by law enforcement worldwide between 2022 and 2023. Methamphetamine use is of particular concern in East and South-East Asia, where nearly three-quarters of people seeking treatment for any substance use disorder are seeking care for methamphetamine use specifically (United Nations Office on Drugs and Crime, 2025).

### 1.2 Molecular mechanisms of stimulant action

Stimulant effects are largely mediated by direct interactions with monoamine signaling machinery, specifically the reuptake transporters for dopamine (DAT), norepinephrine (NET), and serotonin (SERT) (Han and Gu, 2006; Fleckenstein et al., 2007; Calipari and Ferris, 2013). Cocaine and methylphenidate primarily inhibit these transporters, reducing reuptake and increasing extracellular concentrations of dopamine and norepinephrine. Amphetamine-type stimulants additionally enter monoaminergic neurons through transporters; once inside the cytoplasm, they enter synaptic vesicles via vesicular monoamine transporter (VMAT) and induce an intravesicular pH change to expel neurotransmitters from these synaptic vesicles (Han and Gu, 2006; Fleckenstein et al., 2007). This may drive non-exocytotic release via reverse transport through DAT, though *in vivo* evidence suggests that this effect is inconsistent and secondary to DAT inhibition and enhancement of exocytotic release (Ramsson et al., 2011a; Ramsson et al., 2011b; Covey et al., 2016). Stimulants additionally have many secondary mechanisms of action that reinforce these effects. For instance, inhibition of monoamine oxidase (MAO) slows cytoplasmic degradation of monoamines, increasing the amount of monoamines available for release (Sulzer et al., 2005).

Stimulants engage the brain’s reward and reinforcement circuitry, producing acute euphoria (“reward”) and strengthening the drive to use again (“reinforcement”). Reinforcing effects are hypothesized to be driven largely by increased dopamine signaling within the mesolimbic system (Willuhn et al., 2010), particularly at projections from the ventral tegmental area to the nucleus accumbens, which itself promotes learning about drug-associated cues. With repeated exposure, reward systems adapt to the acute effects of the drug, with affected neurons showing reduced sensitivity to future dopaminergic inputs. When the drug is no longer present, these adaptations may persist and contribute to withdrawal symptoms, which include reduced sensitivity to natural rewards (Koob and Bloom, 1988). On a molecular level, this is linked to reduced availability of D2-type dopamine receptors, particularly in the orbitofrontal gyrus and cingulate (Wang et al., 2012; Moeller et al., 2018). This reduction has been repeatedly observed in former stimulant users, even well into abstinence (i.e., beyond acute withdrawal) (Volkow et al., 1993); relevant to this review, sleep deficits may contribute to this effect (Hollander et al., 1990).

In parallel, stress-related pathways involving corticotropin-releasing factor, norepinephrine, and dynorphin, along with regions such as the habenula, may contribute to negative emotional states during abstinence, and these negative states drive resumption of use (Hikosaka, 2010; Koob et al., 2014). Orexin (hypocretin) has additionally emerged as another neuromodulator implicated in stimulant use disorder. Orexin-releasing neurons are located exclusively within the hypothalamus and project widely to regions such as the ventral tegmental area and nucleus accumbens, positioning this system to modulate drug cravings and reward. Orexin neurons also interact with CRF-containing stress circuits (Winsky-Sommerer et al., 2004; Navarro et al., 2015; Fragale et al., 2021; Capó et al., 2025). These varied molecular actions mean that stimulants have behavioral consequences spanning many timescales and neural systems, contributing to the complexity of SUD.

### 1.3 Clinical characteristics and treatment of stimulant use disorder

Stimulants exert strong acute somatic effects including tachycardia, hypertension, hyperlocomotion, and agitation. At higher concentrations, psychostimulant toxicity can induce hyperthermia and cardiovascular or cerebrovascular complications (Clinical Guideline Committee Members et al., 2024). Some of these effects have been hypothesized to be driven by stimulant-induced amplification of norepinephrine signaling. This increases sympathetic nervous system activity to cause tachycardia and vasoconstriction, and in turn, agitation, hypertension, hyperthermia, and a significantly increased risk for cardiac death (Docherty and Alsufyani, 2021).

Despite a host of negative symptoms, there is no FDA-approved pharmacological treatment for SUD, which may be explained by the complex effects of stimulants on reward circuitry across multiple neurotransmitter systems. Although a wide range of pharmacologic agents (dopamine receptor agonists, antipsychotics, antidepressants, and anticonvulsants) have been tested, systematic reviews of these interventions have failed to identify a consistent benefit of any of these treatments (Sangroula et al., 2017; Ronsley et al., 2020; Acheson et al., 2023). Newer pharmacologic agents have shown modest promise; for example, extended-release naltrexone combined with bupropion produced higher response rates than placebo in a randomized trial for the treatment of methamphetamine use disorder, although overall response rates remained low (Trivedi et al., 2021). Mirtazapine has also been shown to modestly reduce methamphetamine use (Colfax et al., 2011; McKetin et al., 2026), possibly because its actions on serotonin and histamine receptors help address the insomnia and anxiety commonly seen in methamphetamine use disorder (Graves et al., 2012; Rush et al., 2025). Finally, orexin antagonists also show promise for treating SUD; one such antagonist, suvorexant, is already FDA-approved for insomnia (Center for Drug Evaluation and Research, 2025). Evidence for its use in SUD remains mixed, with multiple randomized trials currently ongoing (Stoops et al., 2022; Webber et al., 2026).

Outpatient treatment of SUD in the U.S. is relatively low, with only 19.7% of people age 12+ diagnosed with methamphetamine use disorder seeking treatment in 2024 (Substance Abuse and Mental Health Services Administration, 2025). Due to the lack of pharmacological treatments, SUD is instead treated primarily with psychosocial and behavioral interventions such as contingency management, which aims to reinforce positive behaviors (e.g., abstinence) through rewards like prizes, cash vouchers, or conditional employment opportunities; when used in conjunction with cognitive behavioral therapy and community support, such programs can improve SUD outcomes. Unfortunately, social, legal, and financial barriers make widespread implementation of contingency management programs difficult (Clinical Guideline Committee Members et al., 2024). Additionally, these programs would likely have improved efficacy if combined with a targeted pharmacological agent, as has been demonstrated in both opioid use and alcohol use disorders (Cohen et al., 2022; Lee et al., 2024).

SUD carries major morbidity and mortality risk (Farrell et al., 2019). Common comorbidities such as mood and anxiety symptoms, ADHD, polysubstance use, and an elevated risk of psychotic symptoms can complicate clinical presentation, reduce treatment engagement, and worsen outcomes. Medical comorbidities are also common, arising from injection-related risks, impaired judgment, and adverse social conditions; this contributes to increased risk of HIV, viral hepatitis (Colfax et al., 2010), aforementioned heart disease, and other chronic health complications in stimulant-dependent populations. Sleep complaints, reported in 66.5% of patients in one detoxification cohort (Grau-López et al., 2020), also fit within this broader comorbidity profile.

### 1.4 Introduction to human sleep

Sleep is a necessary physiological state of prolonged immobility and reduced sensory awareness found across the animal kingdom. Current public health guidelines recommend between 7 and 9 h of sleep each night, though about half of the U.S. population falls under this benchmark (Liu S.et al., 2016). This is of great public health concern because the sleeping brain performs several critical functions to maintain health and cognitive ability (Baranwal et al., 2023). For instance, sleep greatly enhances the consolidation of declarative, emotional, and procedural memory by increasing neuroplasticity (i.e., synaptic pruning and strengthening) and decreasing neuroinflammation (Smith, 2001; Tononi and Cirelli, 2014; Blanco et al., 2015; Palagini et al., 2022; Yuksel et al., 2025), making sleep beneficial for emotional regulation and the learning of adaptive behavior (Zhang et al., 2019; Tomaso et al., 2021). Sleep also contributes to metabolic maintenance, as during sleep metabolic rate declines (Saper et al., 2010; Schmidt, 2014; Schneider, 2020) while glial cells facilitate removal of metabolic waste (Yankova et al., 2021).

Beyond these positive functions, sleep is also protective against many forms of mortality. Tiredness, whether due to exogenous sleep disturbance, chronic fatigue, or inconsistent sleep schedules (e.g., due to shift work), is associated with impaired vigilance and memory, increased risk of substance use, and increased risk of death in motor vehicle accidents (Connor et al., 2001; Owens and Weiss, 2017; Grandner and Fernandez, 2021; Moreno, 2025). Supporting the broad importance of sleep for human health and disease, a burgeoning body of research indicates bidirectional relationships between sleep disorder and more severe health issues, including (but not limited to) mood disorders (Rao et al., 2009; Staner, 2010; Jaussent et al., 2011; Dauvilliers et al., 2013; Tomaso et al., 2021; Redeker et al., 2022; Sunwoo et al., 2022), heart disease (Redeker and Stein, 2006; Johansson et al., 2010; Piamjariyakul et al., 2021; Redeker et al., 2022), obesity (Chaput et al., 2023), and substance use disorders (Teplin et al., 2006; Pasch et al., 2012; Dolsen and Harvey, 2017; Drazdowski et al., 2025). Due to the cognitive benefits of sleep and the exacerbating effect of sleep loss on existing health conditions, the high prevalence of sleep disorder across the general population and among substance users specifically is of great concern.

Since the 1950s, sleep in humans has primarily been studied using polysomnography (PSG), which quantifies several physiological parameters including cardiovascular activity, muscle activity including eye movement, and brain activity (as measured by electroencephalogram, EEG). Such studies have revealed that human sleep follows a predictable architecture that consists of a series of roughly 90-min cycles during which the brain switches between rapid eye movement (REM) and non-REM (NREM) sleep (Jafari and Mohsenin, 2010). We will not provide an in-depth review of human sleep architecture in this review, as the study of *Drosophila* sleep architecture is still in its infancy and findings cannot yet be applied to mammalian sleep staging (van Alphen et al., 2021; Abhilash et al., 2026), but note that many studies that will be referenced below find that drugs of abuse induce deficits specifically in REM sleep.

### 1.5 Neural basis and regulation of sleep

As described by the widely successful two-process model, sleep is primarily regulated by two drives (Borbély, 1982; 2022). First, the circadian drive functions to initiate sleep at a consistent time each day (“circa-diem,” ∼24 h). An intact circadian rhythm helps animals organize behaviors in accordance with exogenous rhythms (light, temperature, food availability). These rhythms are maintained by so-called pacemaker cells, which are found in the suprachiasmic nucleus (SCN) of the hypothalamus in mammals (Silver and Schwartz, 2005; Hastings et al., 2018). As will be explored in Section 3, pacemaker cells display oscillatory transcriptional feedback loops, meaning these rhythms are durable even in the absence of external time cues. However, exogenous cues called zeitgebers are important for aligning these internal genetic rhythms with external signals, helping animals maintain regular daily behavior despite (e.g.) seasonal shifts in sunup/sundown time. Light is a potent zeitgeber that acts via melatonin signaling (Zisapel, 2018; Mahoney and Schmidt, 2024), but other cues, including food, social activity, locomotion, and drugs of abuse, can function as zeitgebers even in the absence of the SCN (Mistlberger and Rechtschaffen, 1984; Mistlberger and Skene, 2004; Kosobud et al., 2007).

Sleep timing is also regulated by the homeostatic drive, which ensures that sleep duration meets physiological demands. This process can be intuitively felt after sleep deprivation, which often prompts rebound or “catch-up” sleep to compensate for prior sleep loss; this rebound may even protect against some of the health risks brought upon by chronic sleep restriction (Liu et al., 2024; Li et al., 2026). Several pathways have been proposed to explain the sleep homeostat. A prime molecular candidate is adenosine, a metabolite of ATP that inhibits cortical neurons by binding to inhibitory A1 receptors. This induces cortical inhibition and promotes NREM sleep, during which adenosine is cleared from the brain; in fact, the amplitude of slow-wave EEG during deep NREM sleep is one of the clearest correlates of homeostatic sleep pressure in mammals. Adenosine also excites neurons in specific tissues (e.g., the nucleus accumbens) by binding to excitatory A2A receptors, putatively activating sleep-promoting pathways (Ferré, 2010; Huang et al., 2024). On the circuit level, it has additionally been proposed that the sleep homeostat is a side effect of synaptic plasticity. Briefly, during wakefulness, constant sensory input drives synaptic strengthening; sleep thus serves to re-normalize synaptic weights in order to maintain the brain’s ability to adapt to future sensory input, largely mediated by sleep-specific changes in AMPA-type glutamate receptors in the hippocampus and cortex (Tononi and Cirelli, 2014; Cirelli and Tononi, 2022). Relevant to this review, both adenosine signaling and sleep-related changes in glutamate receptor expression have been linked to SUD-relevant behaviors in rodents (Hack and Christie, 2003; O’Neill et al., 2014; Chen et al., 2015).

In evolutionary terms, the fitness benefits of sleep must be weighed against the costs: a sleeping animal cannot complete biologically necessary behaviors like feeding, mating, or predator avoidance. As such, sleep-wake state changes are influenced by action selection circuits in the basal ganglia (Qiu et al., 2010), with dopamine in particular having a strong wake-promoting function. Inhibition of action-selecting dopaminergic neurons projecting from the ventral tegmental area to the nucleus accumbens increases sleep, while activation of those same neurons strongly suppresses sleep (Eban-Rothschild et al., 2016; Mieda, 2017). Dopamine signaling additionally suppresses sleep by inhibiting sleep-promoting GABAergic neurons in the ventrolateral preoptic area, helping consolidate wakefulness (Gallopin et al., 2004). This evidence indicates an important role of dopaminergic signaling in suppressing the sleep state and permitting motivated behavior.

These systems converge on effector circuits that directly control wake and sleep states. Wakefulness is maintained by a network of brainstem nuclei known as the ascending reticular activating system (ARAS). These nuclei release neurotransmitters such as norepinephrine, histamine, acetylcholine, glutamate, and orexin to excite brain the cortex and thalamus, facilitating motor behavior, attention, and memory formation. They additionally inhibit sleep-promoting regions and maintain the awake state; release of orexin in particular serves as a positive feedback loop that strongly maintains ARAS activation (Datta and MacLean, 2007; Saper et al., 2010; Morin et al., 2015; Huang et al., 2024). To induce sleep, inhibitory GABAergic projections inhibit orexinergic and monoaminergic neurons in the ARAS to suppress cortical excitation (Lu et al., 2000; Qiu et al., 2010). GABA also inhibits thalamocortical pathways delivering sensory information to the cortex (Saper et al., 2010), and both GABAergic and glycinergic projections to the spinal cord facilitate inhibition of skeletal muscle. Finally, specific alterations in cholinergic and adrenergic contribute to sleep-specific brain activity (Datta and MacLean, 2007; Morin et al., 2015; Pelayo and Dement, 2017; Huang et al., 2024).

**FIGURE 1:**
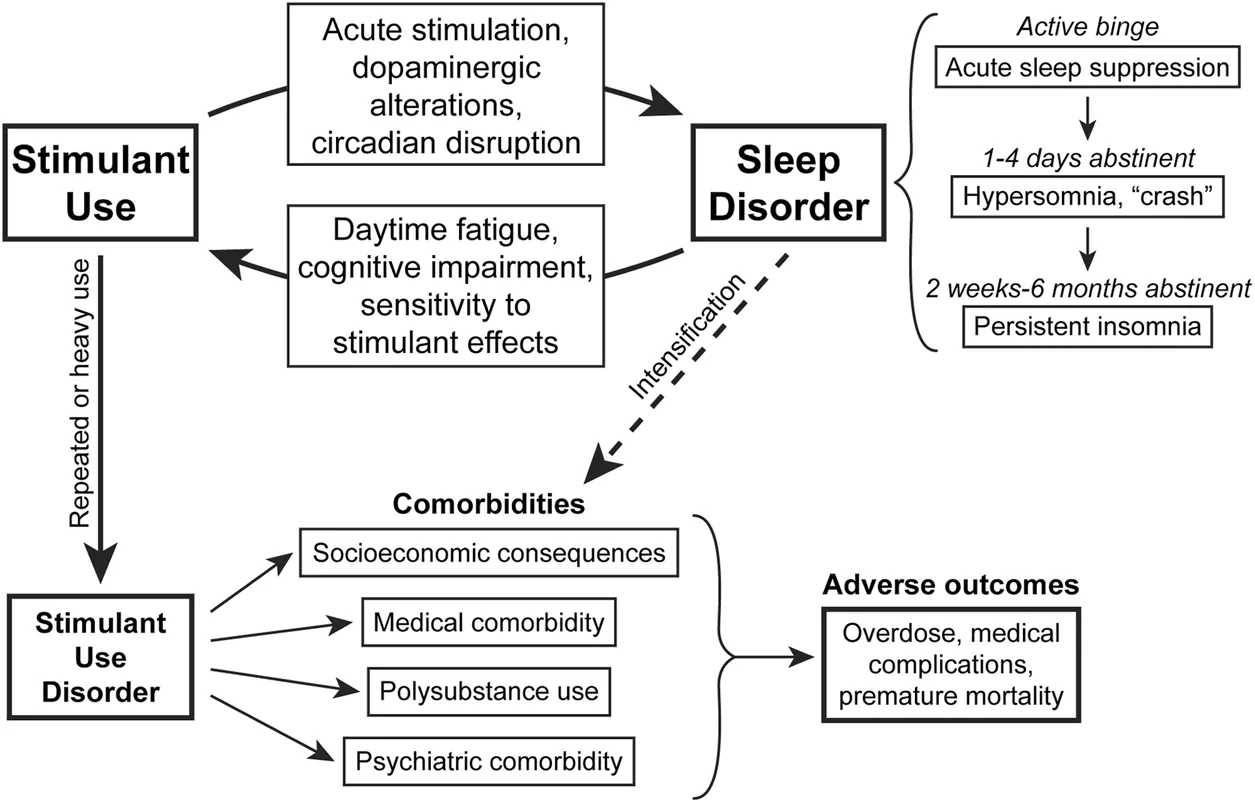
Overview of the bidirectional relationship between stimulant use and sleep disorder, based on information from human studies and clinical observations.

As will be explored further in Section 3, these sleep-regulating systems are deeply conserved across species, with dopamine promoting arousal in many model systems, homeostatic sleep mechanisms identified all across the animal kingdom, and circadian rhythms found even in non-animal life (Campbell and Tobler, 1984). Therefore, non-human animal models are powerful tools for uncovering the neural bases of sleep and sleep-related disease. However, despite decades of empirical research into sleep, widespread recognition of the necessity of sleep for human health, and the ongoing identification of specific neural pathways by which sleep states are regulated and maintained, remarkable gaps in knowledge remain, especially in cases of pathologies that impact sleep regulation. This is of particular concern for the study of SUD because of the reciprocal relationships between sleep disorder, stimulant use, and relapse (Figure 1).

### 1.6 Stimulant use disorder drives prolonged sleep impairment

While SUD is a complex condition with an array of cognitive, emotional, and physiological effects, studies in human stimulant users repeatedly identify persistent sleep difficulties as a core symptom that continues into abstinence. Stimulant use acutely impairs sleep by reducing total sleep time, increasing the number of nighttime awakenings, and increasing latency to fall asleep (Oswald and Thacore, 1963; Rechtschaffen and Maron, 1964; Watson et al., 1972; 1992; Johanson et al., 1999; McGregor et al., 2005; Morgan et al., 2006; Perez et al., 2008). This pattern of suppressed, fragmented sleep follows from the strong release of the wake-promoting neurotransmitters dopamine and norepinephrine.

Crucially, sleep difficulties do not end after cessation of use. After intense or prolonged use, the early abstinence period (1–4 days) is marked by a “crash” consisting of fatigue, increased total sleep time, and daytime drowsiness (Weddington, 1990; Kowatch et al., 1992; Watson et al., 1992; Johanson et al., 1999; Pace-Schott et al., 2005b; 2005b; Morgan et al., 2006; Mahoney et al., 2012; 2014; Wood et al., 2025). Moreover, unlike typical homeostatic rebound sleep, which may be associated with cognitive benefits (Muto et al., 2016; van Alphen et al., 2021; Liu et al., 2024), the “catch-up” sleep reported after a stimulant binge is associated with a decline in cognitive measures (Morgan et al., 2006). In contrast, late abstinence (beginning 1–3 weeks after last use) is characterized by an insomniac phenotype: low total sleep time, increased sleep disturbances, and increased latency to fall asleep (Weddington, 1990; Kowatch et al., 1992; Thompson et al., 1995; Johanson et al., 1999; Pace-Schott et al., 2005a; Pace-Schott et al., 2005b; Morgan et al., 2006; Morgan et al., 2010). Evidence grows sparser beyond 1 month of abstinence, but studies have found deficits including fatigue, decreased sleep time, and increased nighttime awakenings as long as 3 (Rezaei-Ardani et al., 2021) and 6 months after last use (Modarresi et al., 2018; Wood et al., 2025), demonstrating the persistence of these drug-induced behavioral changes even long after any trace of stimulant has left the system.

Unfortunately, the body of human SUD-sleep literature has notable methodological inconsistencies that preclude easy interpretations of the data (Bjorness and Greene, 2021). First, although years of substance use can be measured and controlled for, relevant features such as the intensity, setting, or frequency of stimulant use are harder to quantify. Most stimulant-dependent subjects also take other drugs, most often caffeine, alcohol, cannabis, and/or nicotine, each of which can independently alter sleep (Koob and Colrain, 2020; Song et al., 2020; Chvilicek et al., 2025). Beyond just other substances, comorbidities both social and medical can impact sleep separately or synergistically with effects of stimulant use. Sleep monitoring methodology also varies between studies; for example, self-reported sleep quality (e.g., a survey) can differ greatly from “objective” measurement (e.g., PSG), especially in people with SUD (Morgan et al., 2006; Acheson et al., 2024). Inpatient treatment programs or overnight lab stays for sleep monitoring also introduce confounds such as imposed bedtimes and controlled, quiet environments, which alter sleep compared to more naturalistic designs (i.e., using actigraphy to record sleep in subjects’ home environments). Finally, available studies include mostly or exclusively male subjects. Although males represent the global majority of people with SUD, there are large sex differences in the onset, presentation, progression, and prognosis of SUD, so the relative lack of research on female subjects is concerning (Brecht et al., 2004; Price et al., 2011; Armenta-Resendiz et al., 2024). Altogether, these confounds complicate interpretation of the above studies, but the general trend of short-term hypersomnia followed by long-term insomnia is resilient.

### 1.7 Impact of abnormal sleep on drug use

Large-scale human studies examining substance use writ large identify associations between individuals experiencing sleep difficulties and propensity for substance use in general (Shibley et al., 2008; Brower and Perron, 2010; Pasch et al., 2012; Zavar Mousavi et al., 2024; Dobani et al., 2025; Wang et al., 2025). It should be noted that these data are heterogeneous, as “sleep disturbance” can refer to insomnia, shift-work-related sleep difficulty, polysomnographic sleep deficits, or self-reported sleep trouble, but some findings are nonetheless robust. For instance, in patients with alcohol use disorder specifically, difficulty falling asleep is strongly associated with relapse to alcohol drinking (Foster et al., 1998; Drazdowski et al., 2025). Alcohol use disorder and relapse are also dependent on circadian factors, as these problems are more common in shift workers and people with circadian misalignment (Gamble et al., 2011; Hasler and Clark, 2013; Sharma and Nelson, 2024; Moreno, 2025), and mutations in circadian genes are associated with both alcohol use and sleep problems (Comasco et al., 2010; Kovanen et al., 2010). Stepping beyond alcohol use disorder, the risk of any substance use during abstinence is highest when positive affect is diminished following nights of poor sleep (Lydon-Staley et al., 2017), and reduced executive control mediates these increased cravings (Freeman and Gottfredson, 2018). Finally, correlational data indicate that sleep is protective against relapse in SUD, as patients whose sleep metrics normalized within the first month of abstinence were less likely to relapse to cocaine use (Angarita et al., 2014). This may again be due to the beneficial effects of sleep on executive control, specifically response inhibition (Li et al., 2023), as sleep quality in cocaine-recovering patients predicts cognitive impairments in domains such as vigilance, procedural learning (Morgan et al., 2006), and visual discrimination (Morgan et al., 2008). These deficits are known to decrease retention in outpatient cognitive behavioral therapy for cocaine use (Aharonovich et al., 2006; Lipinska et al., 2015).

Shifting to interventional studies, it is known that experimental sleep deprivation increases initial sensitivity to the subjective and reinforcing effects of stimulants (Roehrs et al., 2004), and the inverse may also be true: alleviating stimulant-induced sleep disorder may improve patient outcomes. For instance, morning administration of the atypical dopamine reuptake inhibitor modafinil can reduce daytime fatigue and improve nighttime sleep in subjects with both sleep disorders (US Modafinil in Narcolepsy Multicenter Study Group, 2000) and stimulant use disorders (Morgan et al., 2010; 2016). This has been found to drive a corresponding decrease in stimulant self-administration and increase in treatment retention in stimulant users without other ongoing substance dependencies (Dackis et al., 2005; McGregor et al., 2008; Anderson et al., 2009; Morgan et al., 2010), though evidence is mixed for its efficacy among stimulant-users in general (Sangroula et al., 2017; Hersey et al., 2021). Therefore, although sleep difficulty increases propensity to resume stimulant use, sleep remains a poorly understood and often uncontrolled mediator of several critical SUD symptoms.

### 1.8 Insights from mammalian models into the intersection of SUD and sleep

Experiments in non-human mammals give insight into specific pathways that may link sleep difficulties to stimulant use. First, rodent studies confirm that DAT is the primary mediator of stimulant effects, as mice with cocaine-insensitive DAT fail to produce typical cocaine-associated behaviors, including conditioned place preference and circling (Chen et al., 2006). Next, stimulant exposure causes lasting changes in dopamine release, reuptake, and reception that persist long beyond drug administration. For example, rat models of cocaine dependence find reduced DA release and reuptake *in vivo* at the end of a self-administration paradigm (Calipari et al., 2014). Furthermore, these rats show reduced metabolism of radiotagged deoxyglucose activity in the nucleus accumbens and in the sleep-relevant dorsal raphe and locus coeruleus areas in the first 48 h of abstinence, indicating reduced functional activity in these regions (Calipari and Ferris, 2013; Calipari et al., 2013). These changes are persistent, as 4 weeks after the start of abstinence from a cocaine self-administration paradigm, rats had enhanced baseline dopamine reuptake in the nucleus accumbens (Alonso et al., 2022), and a single neurotoxic dose of methamphetamine was sufficient to deplete levels of dopamine and serotonin for up to 7 weeks after stimulant administration (Silva et al., 2014). Finally, non-human primate models of methamphetamine or cocaine dependence find reductions in D2-type DA receptor availability in the caudate and putamen for up to 7 weeks after stimulant administration (Groman et al., 2012), with deficits persisting for up to a year of abstinence (Nader et al., 2006; Allen et al., 2023). These alterations in DA signaling machinery have wide-reaching implications for arousal and addiction. For instance, prolonged drug-induced DA signaling may contribute to enhanced plasticity in the nucleus accumbens and prefrontal cortex, facilitating the development of drug dependence (Kalivas, 2007).

Sleep disturbance itself also alters activity in rat dopamine systems, and this generally increases the propensity to develop stimulant dependence. First, selective sleep disruption in rodents increases brain dopamine concentration, particularly in the basal forebrain (Zant et al., 2011). Sleep restriction increases dopamine receptor sensitivity and D2-type dopamine receptor expression in the basal ganglia while decreasing dopamine reuptake (Tufik et al., 1987; Nunes et al., 1994; Andersen et al., 2005). This increases susceptibility to drug conditioning, as in stimulant-naïve rats, two sessions of amphetamine conditioning was sufficient to induce conditioned place preference in acutely sleep-deprived rats, but not in rats with intact sleep (Berro et al., 2018); similar effects have been reported for cocaine, notably implicating orexin receptors (Bjorness and Greene, 2020). Emerging evidence suggests that glial cells may mediate some of these shifts and resulting vulnerabilities to SUD, as astrocytes and microglia in the nucleus accumbens serve as mediators between stress (e.g., stress induced by sleep deprivation) and synaptic changes (Cancela et al., 2025). However, note that the acute sleep restriction paradigms used in these studies generally do not resemble human sleep difficulty, which typically consists of consecutive days or weeks of partially restricted sleep rather than 1–7 days of partial or total sleep deprivation. However, rats experiencing a chronic sleep restriction paradigm meant to mimic human sleep restriction still demonstrated increased subjective value of cocaine and accelerated cocaine self-administration during acquisition (Puhl et al., 2013).

Shifting to rodent models of stimulant abstinence, sleep restriction delayed and dampened extinction of conditioned responses to cocaine (Berro et al., 2014) and methamphetamine (Shahveisi et al., 2022). Inversely, in a mouse model of cocaine abstinence, experimentally enhancing sleep during the light (inactive) period via forced dark-period activity (behaviorally comparable to modafinil-induced consolidation of daytime wakefulness in humans) decreased incubation of cocaine craving; sleep enhancement seemingly counteracted the abstinence-induced accumulation of calcium-permeable AMPA-type glutamate receptors in the nucleus accumbens, potentially reducing excitability in the nucleus accumbens compared to animals that were selectively sleep deprived (Chen et al., 2015; Guo et al., 2022).

Finally, rodent studies elucidate how circadian factors interact with stimulants to influence the progression of stimulant dependence. Circadian periods alter acute responses to stimulants and development of self-administration (Falcón and McClung, 2009); in mice, mutations in circadian genes enhance cocaine reward and place preference, dependent on increased expression of tyrosine hydroxylase and dopamine neuron excitability (McClung et al., 2005). Stimulants reciprocally influence circadian rhythms, as a daily midday cocaine exposure induces circadian phase advance in rats, meaning that in total darkness these rats developed a circadian period shorter than 24 h (Glass et al., 2012). Interestingly, METH may instead induce circadian phase delay that persists even into withdrawal (Honma et al., 1986). Methamphetamine additionally induces a second circadian activity cycle, separate from the ∼24-h circadian clock and endogenous pacemaker neurons, as this stimulant-induced phasic activity can be induced even in mice lacking the circadian *clock* gene and deprived of external day/night cues (Masubuchi et al., 2001; Tataroglu et al., 2006). Genetically, METH induces transcription of the circadian *per1* gene in the caudate and putamen, dependent on D1 receptor function, and this may serve as a mechanism for locomotor sensitization to METH, as well as a means by which METH can alter circadian periods (Nikaido et al., 2001). Epigenetic and glial factors regulating the circadian cycle likely influence these interactions as well (for reviews, see Freyberg and Logan, 2018; Saad et al., 2021).

This work supports the conclusion that homeostatic and circadian sleep disorders are mechanistically linked to the development and reinstatement of substance use. However, gaps in knowledge remain regarding specific molecules, neural populations, and time courses of these effects, and studies in simpler model systems will be useful in filling these gaps.

## 2 Approaches to studying SUD and sleep in *Drosophila melanogaster*

### 2.1 *Drosophila* as a neuroanatomical model system

The vinegar fly *Drosophila melanogaster* is a well-established invertebrate model of the genetic, molecular, and neural bases of complex behaviors (Kaun et al., 2012). As a model, flies are useful because they have short generation times (around 12 days), low costs, scalable behavioral assays, and widely-available genetic tools. The fly genome is fully sequenced, with a genome size of about ∼200 megabases organized across four chromosome pairs, making gene identification and manipulation highly efficient compared with many vertebrate systems (Bosco et al., 2007). Additionally, it is estimated that 75% of human disease-related genes have conserved counterparts in *Drosophila* (Reiter et al., 2001). This genetic similarity, combined with a nervous system that supports behaviors such as learning (Quinn et al., 1974), sensorimotor integration (Seelig and Jayaraman, 2011), and social interaction (Chen et al., 2002), makes the fly a powerful tool for dissecting mechanisms of neural function and behavioral regulation.

Core neurotransmitter systems, particularly the monoamines, are conserved between mammals and flies. Flies possess dopamine and serotonin, and although they lack norepinephrine, octopamine likely serves as a functional analogue (Roeder, 1999). Synthesis pathways are also highly conserved. For example, the fly gene *ple* encodes the enzyme Tyrosine Hydroxylase (TH), which is the rate-limiting step for dopamine synthesis in both humans and flies (Neckameyer and Quinn, 1989). Serotonin synthesis is also conserved, with the *Drosophila* tryptophan hydroxylase (Trhn) serving as the ortholog to mammalian TPH (Coleman and Neckameyer, 2005). Finally, tyramine beta hydroxylase converts tyramine into octopamine and is highly homologous to the mammalian enzyme dopamine beta hydroxylase, which is itself required for NE synthesis (Monastirioti et al., 1996). The storage, release, and transport of these neurotransmitters are also managed by conserved proteins; for example, mammals utilize two versions of the vesicular monoamine transporter (VMAT) to load synaptic vesicles, and the *Drosophila* gene *dVMAT* is a single ortholog that serves the same function (Greer et al., 2005). Additionally, the dopamine transporter is a major regulator of extracellular dopamine in both flies and mammals. The *Drosophila* dopamine transporter (dDAT) shares key molecular and functional properties with mammalian catecholamine transporters and is also a molecular target of stimulants (Pörzgen et al., 2001).

Monoamines have functionally similar roles in the regulation of behavior across species. As in mammals (Giros et al., 1996; Wise, 2004), dopamine in *Drosophila* is linked to locomotor activity (Yellman et al., 1997), learning (Zhang et al., 2008; Waddell, 2010; Aso et al., 2014), arousal (Kume, 2006; Ueno et al., 2012), and behavioral responses to drugs of abuse (Chang et al., 2006; Ojelade et al., 2019). Serotonin has been implicated in stress responses (Hu et al., 2020) and depression-like states in *Drosophila* (Ries et al., 2017), supporting the idea that serotonergic regulation of affective behavior is evolutionarily conserved (Cools, 2008). Octopamine is important for arousal in flies and has been implicated in locomotor control (Hardie et al., 2007; El-Kholy et al., 2022), sleep regulation (Crocker and Sehgal, 2008), aggression (Hoyer et al., 2008), and memory (Selcho, 2024), similar to NE in mammals (Aston-Jones and Cohen, 2005). These similarities in neurotransmitter synthesis and function make flies a mechanistically valid model for studying the neuropsychopharmacology of drugs of abuse (Narayanan and Rothenfluh, 2016; Philyaw et al., 2022).

In addition, some fly and mammalian brain structures, while morphologically different, are thought to fulfill analogous behavioral functions and may have shared evolutionary roots (Strausfeld and Hirth, 2013; Borst and Helmstaedter, 2015). For example, the insect central complex and vertebrate basal ganglia have common roles in action selection, motor control, and other complex behaviors. In *Drosophila*, disruption of the central complex circuitry results in parkinsonism-like defects, such as ataxia, hesitancy, and sleep disturbances (Strauss, 2002; Ueno et al., 2012), similar to basal ganglia disruption in mammalian models (Takakusaki et al., 2004; Qiu et al., 2010). This functional analogy is supported by shared stem cell lineages and developmental genetic cascades, similarities in structural arrangement of these structures, and a shared role of dopamine in regulating activity in these structures (Strausfeld and Hirth, 2013).

Of course, the *Drosophila* model is limited in the extent to which every stimulant-relevant neurotransmitter system can be explored in-depth. For instance, as discussed in Section 1, orexin (hypocretin) has been widely implicated in motivated behavior, sleep/wake regulation, and cocaine responses (Saper et al., 2001; España et al., 2010; Mensen et al., 2015; Shaw et al., 2017; Martin-Fardon et al., 2018; Briggs et al., 2019), but studying this neuropeptide in *Drosophila* is likely infeasible, as in contrast to the loose equivalence between norepinephrine and octopamine, there seems to be no *Drosophila* analogue for orexin. It is worth noting that other invertebrates express allatotropins, which have been shown to mediate similar functions as orexins and whose receptors bear high structural homology to orexin receptors, but these proteins have not yet been identified in *Drosophila* (Hewes and Taghert, 2001; Vanden Broeck, 2001; Alzugaray et al., 2019; Konopińska et al., 2024).

### 2.2 Genetic manipulation approaches to study *Drosophila*

One of the most widely used approaches for manipulating genes and neurons in flies is the Gal4/UAS binary expression system, which allows transgenes to be expressed in defined tissues or cell populations. In this system, a yeast-derived transcription factor Gal4 is expressed under a specific endogenous promoter. Gal4 then triggers expression of a construct downstream of an upstream activating sequence (UAS) (Brand and Perrimon, 1993). Given the large number of Gal4 drivers available from public collections such as a Bloomington *Drosophila* Stock Center, neuronal targeting ranges from the whole brain to clusters as small as tens of neurons. The expressed construct is similarly versatile: it could be a gene to increase copy number, an RNA interference (RNAi) construct for knocking down gene expression, a fluorescent reporter, or a tool to manipulate neuronal activity. Differentially manipulating and/or monitoring distinct neural populations at the same time can be achieved by combining the Gal4/UAS system with the analogous LexA/LexAop system (Lai and Lee, 2006).

The high spatial specificity of the Gal4 system and its derivatives is highly useful for the study of sleep and stimulants because certain cell types in the *Drosophila* brain are constituted by tens of neurons or fewer (Zheng et al., 2018; Dorkenwald et al., 2024; Schlegel et al., 2024). For example, the PPL1 cluster of dopamine neurons has about 12 neurons per brain hemisphere (Mao and Davis, 2009), and of those neurons, just 1–2 provide behaviorally relevant input to a sleep circuit (Liu et al., 2012; Hulse et al., 2021). To target such sparse populations, the split-Gal4 system restricts expression to cells where two separate Gal4 expression patterns overlap (in boolean logic, “Gal4-A AND Gal4-B”); in contrast, expressing Gal4 in combination with its transcriptional repressor Gal80 prevents expression of the UAS in certain subsets of cells or tissues in the Gal4 expression pattern (in boolean logic, “Gal4 AND NOT Gal80”) (Luan et al., 2006). In addition, temporal control can be achieved using temperature-sensitive constructs such as Gal80^ts^, allowing gene expression or neuronal manipulation to be limited to specific developmental/adult stages or time windows (McGuire et al., 2004), for example, the exposure or recovery phase of stimulant exposure.

Targeted genome editing with CRISPR-based approaches has further expanded the genetic toolkit in *Drosophila*. In the CRISPR/Cas9 system, the DNA-cutting enzyme Cas9 is directed to a specific genomic sequence by a single-guide RNA (sgRNA), allowing the generation of targeted gene knockouts (Bassett et al., 2013; Gratz et al., 2013). Other CRISPR-based approaches, such as the dCas9-VPR system, use sgRNAs to direct inactive Cas9 fused to transcriptional activation domains to a target gene, increasing endogenous gene expression without cutting the DNA (Lin et al., 2015). In both knockout and activation approaches, Cas9 or dCas9-VPR expression can be controlled using the Gal4/UAS system, allowing gene manipulation to be restricted to specific tissues and cell populations.

### 2.3 Monitoring and manipulating neurons in *Drosophila*

A wide range of constructs can be used to monitor and manipulate neuronal activity in *Drosophila*. When combined with binary expression systems discussed above, these tools allow researchers to record, activate, or silence activity in defined neuronal populations while measuring behavioral outputs.

Neural monitoring has classically been accomplished by directly measuring electrical activity in neurons with patch-clamp electrophysiology. Genetically defined neuron populations can be targeted visually by expressing GFP under a specific Gal4 (Gu and O’Dowd, 2007), and recordings can be performed in multiple neurons simultaneously to explore connectivity (McCarthy et al., 2011), though these approaches are challenging due to the small size of *Drosophila* neurons (∼4 µm).

Newer optogenetic approaches using genetically-encoded calcium indicators (GECIs) such as GCaMP circumvent many issues of electrophysiological methods, enabling non-invasive real-time optical measurement of neural activity in Gal4-defined populations (Melom et al., 2013; Chen et al., 2026). The acuity of these constructs is quite remarkable, as 5^th^- and 6^th^-generation GcaMP constructs have high enough resolution to detect single action potentials and vesicle release events with near-perfect correspondence to electrophysiological methods (Astacio et al., 2022), while 8^th^-generation constructs can quantify calcium transients in even fast-spiking neurons on timescales relevant to neural computation and integration (Zhang et al., 2023). The CaLexA system similarly measures neuron activity via calcium signaling but trades away GcaMP’s high temporal resolution for a reporter that accumulates with sustained neural activity. This timescale of hours or days may be more appropriate for studying the long-term changes induced by stimulant exposure (Dolmetsch et al., 1997; Masuyama et al., 2012; Guo et al., 2016; Guo et al., 2017). CaLexA can also be used to drive expression of FLP constructs or the Gal4/UAS system to induce long-term changes in gene expression in neural populations defined by both activity and genetic identity (Guenthner et al., 2013), e.g., enabling researchers to label cells that are hyperactivated after a stimulant exposure.

In addition to merely monitoring activity, neuronal excitability can be directly altered in Gal4-defined cell populations. Synaptic vesicle release can be transiently suppressed or induced using thermogenetic or optogenetic tools (e.g., dTrpA1, shibire^ts^) (Kitamoto, 2001; Boyden et al., 2005) or ATP-sensitive cation channels (Lima and Miesenböck, 2005; Klapoetke et al., 2014). Ion-channel-based effectors (e.g., Kir, NaChBac, and CaMKII) can persistently increase or decrease neuronal excitability without direct activation or inhibition of neurotransmitter release (Ren et al., 2001; Park et al., 2002; Hodge, 2009), more closely resembling the graded effects of neuromodulators on cell output. Together, tools such as these allow precise manipulation of neural activity within the adult fly brain. In the context of SUD and sleep research, defined neuronal populations (e.g., the small number of dopaminergic neurons) can thus be selectively manipulated, allowing researchers to determine how cell-specific activity contributes to stimulant-induced behaviors and sleep.

**FIGURE 2:**
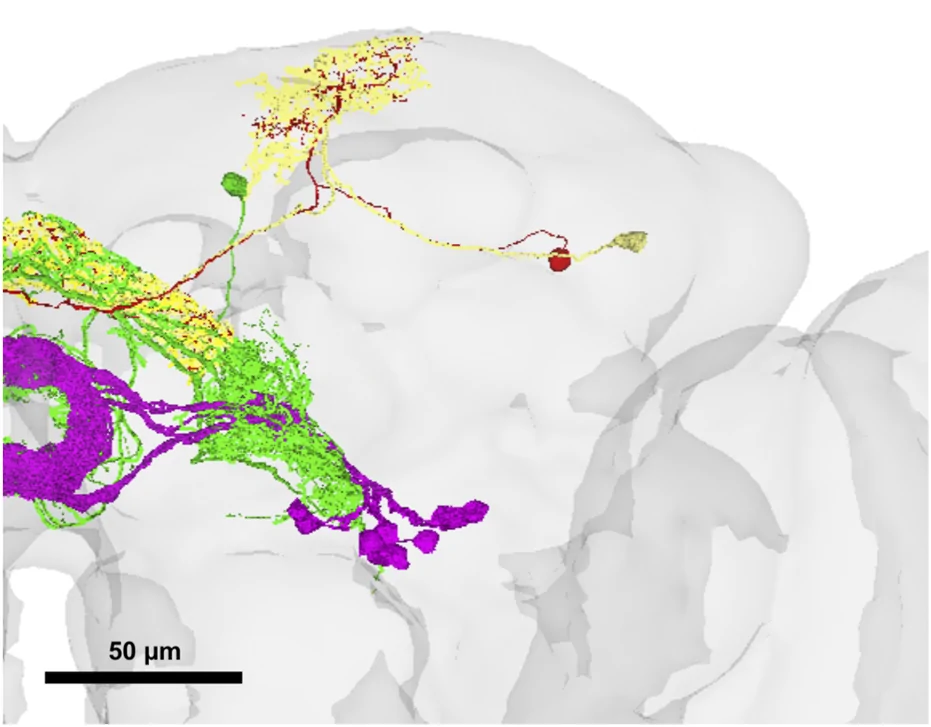
FlyWire reconstruction of one hemisphere of a putative homeostatic sleep circuit in the *Drosophila* central complex, based on morphology and connectivity of implicated neurons. Yellow: A bilateral pair of wake-promoting dopamine neurons have cell bodies in the PPL1 cluster and synapse on axon terminals in the dFB (Liu et al., 2012). FlyWire cell type FB7B neurons displayed. Red: A subset of dFB neurons in the 23E10-Gal4 expression pattern are sleep-promoting when activated (Liu et al., 2012) and receive wake-promoting inhibitory dopamine inputs from PPL1 DA neurons (Pimentel et al., 2016; Donlea et al., 2014; Donlea et al., 2018). FlyWire cell type FB7K neurons displayed. Green: Helicon neurons permit locomotor behavior in response to sensory input during wake and are inhibited by dFB neurons during sleep (Donlea et al., 2018). FlyWire cell type ExR1 neurons displayed. Magenta: Sleep-relevant neurons in the EB receive input from helicon neurons; they have previously been identified as either R5 or R2 neurons. These neurons increase their activity and synapse density after sleep deprivation to encode sleep need (Liu Y. et al., 2016; Donlea et al., 2018; Raccuglia et al., 2019). FlyWire cell type ER5 neurons displayed (Zheng et al., 2018; Dorkenwald et al., 2024; Schlegel et al., 2024).

A unique advantage of *Drosophila* over more complex model systems is the existence of a connectome. Volume reconstruction was performed on electron micrographs of over 7000 40 nm sections, then a consortium of researchers and machine learning algorithms reconstructed and annotated nearly 140,000 neurons and their 54.5 million synapses (Zheng et al., 2018; Dorkenwald et al., 2024; Schlegel et al., 2024). A male connectome with ∼166,000 neurons from the brain and ventral nerve cord has also been completed and annotated, but the publication is not yet peer-reviewed (Berg et al., 2025). This dataset can be compared with existing imaging and functional connectivity data to construct putative maps of neural circuits (e.g., Figure 2).

Relevant for the study of neural circuits, these maps include upstream and downstream synapses and neurotransmitters or neuropeptides putatively released by each neuron. This enables *in silico* predictions about the effects of perturbations–e.g., sleep deprivation, sensory stimuli, and/or drug consumption, each represented as changes in the activity of a set of neurons–on neuron activity on the rest of the circuit. For one example of this, open-source software instantiating the connectome into a leaky-integrate-and-fire model of neural circuits was used to predict the impact of taste neurons on neurons controlling feeding initiation (Shiu et al., 2024); similar neural network models have also been used to generate testable hypotheses within the fly visual system (Lappalainen et al., 2024). For studying SUD, the same software could be used to efficiently perform *in silico* experiments on the effects of altered excitability of different classes of monoaminergic neurons on sleep neurons. However, existing models are less suitable for modeling longer-term effects of DA signaling, specifically synaptic plasticity and altered gene expression.

### 2.4 Monitoring sleep in *Drosophila*

Since the turn of the century, *Drosophila* sleep has been defined as a bout of inactivity lasting 5 or more minutes. This simple behavioral definition meets common hallmarks of a sleep state observed across the animal kingdom, including species-specific posture, rapid reversibility, reduced responsiveness to external stimuli, and regulation by both circadian and homeostatic factors; the validity of this state as a sleep state will be elaborated in more detail in Section 3 and Box 1.

**BOX 1:** Drosophila as an invertebrate model of sleep: face validity

In order for a behavioral state in a given animal to be considered a sleep state, that state must satisfy certain criteria (Campbell and Tobler, 1984; Hendricks et al., 2000b; Shaw, 2003). Data from nearly three decades of research demonstrate that *Drosophila* sleep (as defined as any bout of inactivity lasting 5 or more minutes) meets these requirements.		
Rapidly reversible behavioral quiescence	Voluntary movement is diminished, but unlike other brain-mediated quiescent states (e.g., coma, hibernation, or paralysis), this state can be exited in seconds to minutes.	Flies enter periods of inactivity lasting minutes to hours, during which they are largely immobile, save for respiration-related abdominal pumping, proboscis extension or protraction, and minor twitches in the abdomen or extremities; these minor movements do not seem to be evoked by anything in the environment. Intense stimulation can take flies out of this state within seconds (Hendricks et al., 2000a).
Circadian regulation	This state tends to occur during specific phases of the animal’s circadian period.	Inactivity states dominate at night, though flies (especially males) also display prominent “siesta” bouts of inactivity during the day. Inactivity bouts shorter than 5 min occur most often during the morning and evening feeding periods, and they rarely occur during the first sleep bout of the night. This patterning persists even in the absence of external cues and is reliant on genetic clocks in dedicated neurons (Hendricks et al., 2000a; Blum et al., 2018; Abhilash and Shafer, 2024; Abhilash et al., 2026).
Homeostatic regulation	Preventing an animal from entering this state will increase its propensity to enter this state in the near future.	Applying an intermittent mechanical stimulus that deprives flies of this putative sleep state causes an increase (“rebound”) in these inactivity bouts on the following night (Hendricks et al., 2000a; Huber et al., 2004; Donlea et al., 2014; Abhilash et al., 2026).
Stereotypical posture	Each species has a distinct sleep posture, typically one that reduces sensory input and requires little skeletal muscle effort.	Flies in an inactivity bout sit closer and more parallel to the supporting surface than active flies (Hendricks et al., 2000a).
Elevated arousal threshold	Behavioral responses to stimuli may be delayed or require a greater intensity or duration of the stimulus.	Flies in a prolonged inactivity bout do not respond when active flies collide with them, and a more intense or higher-frequency stimulus is required to evoke a locomotor startle response from inactive flies (Hendricks et al., 2000a; Vienne et al., 2016).
Neural function	This state is mediated by changes in brain activity.	Flies in this putative sleep state display altered local field potentials, neurotransmitter release, and calcium levels; unlike brain activity in an active state, brain activity in this rest state is uncoupled from visual stimuli. Drugs or manipulations that affect sleep-relevant neurons or neurotransmitter systems (e.g., caffeine, gaboxadol) can either enhance or suppress sleep accordingly (Hendricks et al., 2000a; Nitz et al., 2002; Swinderen et al., 2004; Donlea et al., 2011; Bushey et al., 2015; Yap et al., 2017; Raccuglia et al., 2019).

This behavioral definition means that sleep analysis in *Drosophila* largely relies on activity monitoring. In brief, flies are individually housed in small glass cuvettes with food at one end; an infrared beam intersects the middle of the tube. As an active fly moves, it disrupts this beam, producing a signal that is recorded on a computer (Hamblen et al., 1986); an absence of beam breaks indicates sleep. This method is surprisingly sensitive despite its simplicity, as active flies were found to cross the infrared beam in 98% of 5-min bouts (Shaw, 2003), and video tracking produces similar estimates of sleep (Donelson et al., 2012), though such correlations have not yet been demonstrated for flies under the influence of stimulants. Importantly, infrared light does not interfere with fly rhythmicity or behavior (Frank and Zimmerman, 1969). This hardware is now commercially available as the *Drosophila* Activity Monitor (DAM) system (Trikinetics). The broad use of the DAM has led to a myriad of open-source analysis software and customizable hardware relevant for the study of SUD and sleep. For example, a motor or vortexer can be used to selectively deprive flies of sleep (Shaw et al., 2002; Chowdhury et al., 2023), and DAMs can be housed in an incubator to enable light- or temperature-driven changes in gene expression or neural activity using genetic methods described above (Vecsey et al., 2024). These relatively simple techniques enable high-throughput analysis and manipulation of sleep parameters relevant to human sleep disorder, including sleep time, number and duration of sleep bouts, sleep onset latency, and sleep rhythmicity (Shaw, 2003; Chiu et al., 2010; Grandner and Fernandez, 2021; Sitaraman et al., 2024).

Despite the convenience of the DAM, there are obvious limitations in reducing behavioral analysis to a binary state of activity vs. inactivity. Because of this, video analysis has been used for decades (Hendricks et al., 2000a), though it has only recently become accessible at scale due to advancements in computational power, data storage, behavioral analysis techniques, and the integration of machine learning technology to enhance behavioral annotation (Donelson et al., 2012; Geissmann et al., 2017; Mathis et al., 2018; Revel et al., 2025). These methods enable detection of finer-grained behaviors such as postural adjustments or movements in specific appendages. For example, periods of repeated proboscis extension during fly sleep are correlated with reduced local field potentials and increased arousal threshold, possibly indicating a distinct deep sleep stage in flies (van Alphen et al., 2021). This is relevant to the study of sleep and SUD because stimulant intake in flies is associated with a host of specific behaviors that simple activity monitoring cannot reliably detect, and further study of *Drosophila* sleep architecture (as identified by these micro-movements) may lead to insights on human sleep staging.

### 2.5 Studying stimulant responses in *Drosophila*

*Drosophila* enable the study of stimulant responses across multiple scales, with the body of research spanning from genetic influences to acute drug-induced behaviors to circuit-level mechanisms of drug dependence. Methodologies similarly vary in scope, as research in flies can reveal how stimulant responses compare across genetically variable strains, how stimulants change transcriptomes, and how targeted manipulation of specific genes shape stimulant responses (Highfill et al., 2019; Baker et al., 2021a; Baker et al., 2021b).

There is a high degree of flexibility in how drugs can be delivered, with exposure paradigms varying in route, dose, and duration. Different paradigms are apt for studying different facets of SUD; for example, volatilized cocaine enables fast delivery of a known amount of drug for studying acute sensitivity, sensitization, and intoxication-like responses, while stimulants mixed with food or liquid media enable extended exposures and the study of sleep disruption (Andretic et al., 2005; Walters et al., 2012; Filošević et al., 2018; Karam et al., 2022b). *Drosophila* exposed to stimulants exhibit dose-dependent behavioral responses comparable to those observed in mammals. At lower doses, flies demonstrate stereotypies such as increased grooming and circling, while higher doses drive akinesia, seizure-like activity, and death (McClung and Hirsh, 1998; Yates et al., 2007). These effects can be assessed with behavioral scoring, the DAM system, startle-induced negative geotaxis assays, video-based tracking, or repetitive startle-induced hyperactivity paradigms (McClung and Hirsh, 1998; Bainton et al., 2000; Chang et al., 2006; Lebestky et al., 2009), providing numerous well-validated readouts for stimulant-induced behaviors.

Contrasting with predetermined drug exposures, voluntary intake assays instead allow flies to choose between food with or without drug. Methods like dyed food, capacitance measurements, or capillary feeding tubes enable comparison of the amount of each option interacted with or consumed over the duration of an experiment (Ja et al., 2007; Peru y Colón de Portugal et al., 2014; Ro et al., 2014). Because these methods allow self-directed drug intake rather than forced exposure, they are useful for quantifying preference, avoidance, and changes in consumption over time in response to stimulants across different genotypes and neural manipulations. These are particularly useful for determining the effects of genetic background or genetic manipulations on development of addiction-relevant behaviors, as will be discussed in Section 3.

The *Drosophila* model has limitations for studying SUD. First, voluntary intake assays can be influenced by sensory properties of drugs; for instance, cocaine activates bitter-sensing neurons in flies and contributes to initial drug avoidance (Philyaw et al., 2025). Sensory confounds can be addressed experimentally in some cases, for example, by using altered sensory backgrounds, but they remain an important limitation when interpreting fly drug-consumption phenotypes. More relevant to the study of SUD on the whole, human stimulant use is shaped not only by neural mechanisms and genetic vulnerability but also by subjective drug effects, comorbidities, social context, drug availability, and consequences spanning financial, legal, occupational, and interpersonal domains (Warden et al., 2016; Quednow, 2017; Köck et al., 2022). These sociocultural influences on drug use are difficult or impossible to model directly in flies, so *Drosophila* cannot model the full scope of human SUD. Still, fly assays are suitable for studying specific stimulant-induced behaviors, addiction-relevant phenotypes, and the genetic and neural influences on these critical components of SUD.

## 3 Insights from *Drosophila* into SUD and sleep

### 3.1 Face validity of the *Drosophila* sleep state

Many characteristics of sleep in humans generalize across species, and a sleep state in some form has been identified in every animal in which researchers have looked for one (Campbell and Tobler, 1984; Allada and Siegel, 2008; Hartse, 2011). This ubiquity has broad implications for the functions and origins of sleep that go beyond the scope of this review, but for our purposes, the high degree of conservation in sleep behavior allows for the study of the neural bases of sleeping brains in non-human, even invertebrate, model organisms.

At the start of the 21st century, *Drosophila* was identified as a promising candidate for a simpler animal model of sleep. Flies display predictable reductions in activity levels around the same time each day. Based on quantification of these bouts of inactivity using the methods described in Section 2, a putative *Drosophila* sleep state was defined as any bout of 5 or more minutes of inactivity. This state was found to satisfy common criteria for a face-valid sleep state (Box 1). In the decades since, the model has proved useful in uncovering the biological bases of sleep, in particular the neurotransmitters, neuromodulators, and genes that determine sleep time and the links between circadian and homeostatic sleep processes.

Sleep architecture poses a principal limitation for the study of SUD in flies. In humans and other mammals, the REM stage of sleep is particularly altered during and after stimulant use. This is relevant because REM sleep is critical for consolidation of non-declarative types of memory, including procedural and emotional memory. Additionally, dopamine is heavily involved in the transition from NREM to REM sleep, and REM sleep behavior disorder is the strongest known predictor of the development of synucleinopathies such as Parkinson’s disease, which may indicate a link between dopaminergic motor circuits and circuits coordinating REM sleep (Ackermann and Rasch, 2014; Peever and Fuller, 2017; Girardeau and Lopes-dos-Santos, 2021; Brodt et al., 2023). Unfortunately, *Drosophila* lack identifiable NREM/REM sleep stages comparable to those in mammals. Still, the field is beginning to dissect different stages of *Drosophila* sleep, with recent work proposing special attention be given specifically to proboscis extension sleep (van Alphen et al., 2021) and long-bout sleep (i.e., sleep lasting 60 min or more) (Stahl et al., 2017; Anthoney et al., 2023; Abhilash et al., 2026) as neurophysiologically distinct “deep” sleep stages with high relevance to sleep homeostasis; if different stages of fly sleep are found to have distinct molecular, physiological, or behavioral correlates that are similar to those in human sleep, then perhaps flies could be used to study this aspect of SUD as well.

### 3.2 Circadian regulation of *Drosophila* sleep

One of the key accomplishments in the use of *Drosophila* as a model of sleep and activity regulation lies in the Nobel Prize-winning discovery of the biological bases of circadian rhythms. In wild-type flies, activity-rest cycles are strongly periodic, with flies maintaining 24 h rhythms even in constant darkness. These cycles are dependent on genetic clocks constituted by the *period* and *timeless* genes (aided by *clock* and *cycle*), which drive expression and inhibition of each other in a predictable cycle with robust 24 h periodicity. Mutating these genetic “cogs” results in flies with circadian activity cycles that are either too slow, too fast, or absent altogether (Konopka and Benzer, 1971; Bargiello and Young, 1984; Baylies et al., 1987; Sehgal et al., 1994). These molecular clocks are instantiated in ∼150 clock neurons in the fly central brain (Helfrich-Förster, 2003; Shafer et al., 2006) that are functionally comparable to the pacemaker cells in the mammalian suprachiasmatic nucleus (Hastings et al., 2018). In the years since these discoveries, genetic clocks based on transcriptional feedback have been identified in other organisms, including mammals and even the *Neurospora* fungus, demonstrating the predictive validity of flies in uncovering such deeply conserved systems relevant to sleep (Sangoram et al., 1998; Roenneberg and Merrow, 1999; Koritala and Lee, 2017; Cai and Chiu, 2022).

On a circuit level, dopamine signaling is under circadian control in flies, with brain DA levels peaking in the morning and evening and remaining low overnight. In constant darkness, this day-night difference persists, suggesting that this rhythm is regulated by endogenous circadian rhythms rather than external cues (Gonzalo-Gomez et al., 2012). This day-night DA cycle is likely regulated by distinct clusters of DA neurons that integrate signals from clock neurons and project to locomotor circuits. For example, a subset of PPM3 dopaminergic neurons promotes morning and evening activity (Liang et al., 2019), *fruitless*-positive PAL DA neurons promote morning activity, and a subset of PPL1 DA neurons promotes evening activity (Liang et al., 2023). These connections complement findings in mammals demonstrating the influence of circadian genes on DA neuron excitability.

Clock neurons themselves receive dopaminergic inputs, making them potential sites for stimulant-mediated sleep effects. For example, ten large lateral-ventral neurons (l-LNvs) important for daytime activity peaks have also been shown to increase their excitability and promote activity in response to dopaminergic inputs from a subset of the PPL2 DA neurons (Shang et al., 2008; Shang et al., 2011). Excitability in another pair of LNs that promote morning activity is dependent on light-modulated expression of Dop1R1 (Le et al., 2024). Though these findings are relatively small in scope, they do suggest specific molecular changes that could mediate stimulant intake and circadian disorder.

### 3.3 Homeostatic regulation of *Drosophila* sleep

Work on homeostatic sleep systems in flies has revealed much about the regulation of sleep amount and depth. The genetic mechanisms of sleep homeostasis are less straightforward than the circadian system, but studies in flies have nonetheless identified many genes associated with sleep homeostasis, meaning they impact the degree to which sleep duration changes after some perturbation (i.e., after sleep deprivation or enhancement) (Cirelli et al., 2005; Pfeiffenberger and Allada, 2012; Harbison et al., 2017; Souto-Maior et al., 2020; Shafer, 2025). Notable examples of these genes include the NMDA receptor-encoding gene *dNR1*, which promotes sleep and sleep homeostasis while also regulating long-term plasticity and calcium signaling (Tomita et al., 2015; Tomita et al., 2017; Cai et al., 2025); *nemuri*, which promotes sleep under conditions of high sleep need and is implicated in immune function (Toda et al., 2019); and *fumin*, which was initially identified by chance as a short-sleeping mutant fly with impaired rebound after sleep deprivation (Kume et al., 2005; Kume, 2006) and identified again in a forward screen for atypical sleep behaviors (Wu et al., 2008). The *fumin* gene was found to encode the dopamine transporter, and accordingly *fumin* mutants have chronically elevated dopamine and altered stimulant responses that will be discussed below.

A key homeostatic sleep circuit in flies lies in the central complex (CX), a sensorimotor area of the fly brain that spans the protocerebral bridge (PCB), fan-shaped body (FB), ellipsoid body (EB), and lateral accessory lobes (LAL) (Figure 2). Activation of twenty or so neurons in the dorsal fan-shaped body (dFB) was found to induce sleep in flies. These large-field neurons send inhibitory projections to helicon cells in the CX, which normally permit wakefulness by exciting neurons in the EB. These sleep-relevant EB neurons typically drive motor behavior in response to sensory input (Donlea et al., 2011; Ni et al., 2019; Xie et al., 2019). Neuron activity, synaptogenesis, and network synchronicity in this EB neuron population all increase with sleep drive (Raccuglia et al., 2019), and directly activating these neurons can increase homeostatic sleep drive, creating an increased propensity to sleep that persists even after neuron activation ceases. Inversely, silencing these EB neurons during sleep deprivation blocks homeostatic sleep rebound. These effects are mediated by feedback to the sleep-promoting dFB neurons (Liu S. et al., 2016; Donlea et al., 2018), allowing this recurrent circuit to encode homeostatic sleep behavior by modulating circuits at convergence between sensory inputs and behavioral outputs. The sleep-promoting neurons in the dFB thus functionally resemble GABAergic neurons in the mammalian ventrolateral preoptic in nucleus (VLPO) of the hypothalamus, as both sets of neurons encode sleep need by increasing their activity over a waking bout and sending inhibitory projections to arousal-promoting areas to “gate off” sensory input and motor output, facilitating sleep onset (Pimentel et al., 2016; Donlea et al., 2018).

This circuit is also sensitive to dopamine, providing an interface for stimulants to modulate homeostatic sleep drive. A single pair of PPL1 dopaminergic neurons project to the CX and contact sleep-promoting dFB neurons at their axon terminals. DA inhibits dFB output onto the helicon neurons, disinhibiting the rest of the circuit and suppressing sleep (Liu et al., 2012; Ueno et al., 2012; Pimentel et al., 2016; Ni et al., 2019). This wake-promoting dopamine input exists in tension with sleep-promoting input from lateral posterior clock neurons that release allatostatin-A (Ast-A) and glutamate to activate dFB neurons and promote sleep; simultaneous activation of both inputs fragments fly sleep (Chen et al., 2016; Ni et al., 2019). It is plausible therefore that stimulant exposure acutely activates this circuit to suppress and fragment sleep during exposure, while lasting alterations to this circuit (e.g., in dopamine receptor sensitivity or endogenous neural activity) may persist beyond stimulant exposure to induce the deficits observed in abstinence. Interestingly, astrocytes in the EB have also been found to encode homeostatic sleep need (Blum et al., 2021); given the role of glial cells in mediating stress, neuroplasticity, and inflammatory responses in both flies and mammals, these cells could serve as another axis by which stimulants influence homeostatic sleep circuitry.

The mushroom body (MB) is a region of the fly brain important for associative learning, memory, and internal state representations; like the central complex, it is also involved in sleep regulation. The MB consists of 15 compartments composed of ∼2000 intrinsic neurons (Kenyon Cells, KCs), which receive inputs representing sensory context and stimulus valence from neuromodulatory and neuropeptide-secreting neurons. MB outputs are primarily delivered through 34 output neurons (MBONs) that integrate signals across compartments and directly synapse on targets, including the sleep-relevant FB and LAL in the central complex (Sitaraman et al., 2015a; Scaplen et al., 2021; Suárez-Grimalt et al., 2024).

Specific groups of KCs and MBONs are dedicated to sleep regulation, likely through the FB as an effector. In general, prolonged wakefulness and sleep deprivation decrease activity in most KCs, possibly mediating reductions in behavioral responses to stimuli observed after sleep deprivation (Bushey et al., 2015). However, some populations of KCs and MBONs instead become hyperactive during sleep deprivation; activation of these neurons promotes sleep, and the homeostatic sleep rebound after sleep deprivation can be attenuated by silencing the sleep-promoting MBONs. A separate subset of MB neurons promotes wake, and activity in these neurons strongly decreases during sleep deprivation (Sitaraman et al., 2015a; Scaplen et al., 2021). These sleep-modulating MB neurons are sensitive to DA, as DA release from the PAM cluster activates wake-promoting MBONs in the γ, β, and β′ MB lobes to suppress sleep, dependent on DA receptor expression in KCs and MBONs (Sitaraman et al., 2015b; Driscoll et al., 2021). These DA inputs provide an alternate pathway by which stimulants could impact sleep, and given the role of the MBs in learning, it is also plausible that strong DA signals could induce lasting changes in these sleep circuits.

From the available evidence, it is clear that dopamine acts on many populations of sleep-regulating neurons in the fly brain to suppress sleep and promote active behavior, whether in response to sensory cues or at specific times in the circadian period. This supports the mechanistic validity of flies as a model of dopaminergic sleep modulation. Unfortunately, molecular evidence from flies about stimulant-induced alterations in these circuits is sparse, making it difficult to draw conclusions about how sleep pathways change with stimulant intake. Still, the genetic tools available in flies make it conceptually straightforward to test the effect of possible pathways (e.g., D2-type receptor availability, dopaminergic neuron activity, synapse structure, or glial function) on driving sleep effects.

### 3.4 Insights into SUD from *Drosophila*

Studies in *Drosophila* have provided insight into stimulant-related behaviors at multiple levels, including genetics, neurotransmitters, and neural circuitry. Testing how genetics influence stimulant-related phenotypes is particularly valuable, as SUD is highly heritable, with twin studies reporting heritability as high as 0.72 for cocaine use disorder in some populations (Goldman et al., 2005). Much of this genetic work has been enabled by resources such as the *Drosophila* Genetic Reference Panel (DGRP), which consists of ∼200 fully sequenced inbred lines derived from a single population of flies, providing a resource for testing how natural genetic variation shapes complex behavior (Mackay et al., 2012). Using this resource, voluntary cocaine and methamphetamine consumption and preference were examined across 46 DGRP lines, finding substantial variation between different wild-derived isogenic lines. The genetic polymorphisms associated with these phenotypes were found in or near 725 candidate genes for cocaine and 774 candidate genes for methamphetamine, with effects that differed by sex (Highfill et al., 2019). This approach was later extended using an outbred advanced intercross population derived from 37 selected DGRP lines, in which voluntary sucrose, cocaine, and methamphetamine consumption was measured across 18,000 flies, and the highest-consumers were sequenced and compared with randomly selected controls. Genome-wide analysis identified numerous candidate genes for increased stimulant consumption. From this larger set of candidate genes, 22 were selected for RNAi validation, and knockdown of 17 of these genes altered cocaine or methamphetamine consumption, supporting the functional relevance of these candidate genes (Baker et al., 2021a). Beyond heritable genetic variation, transcriptomic studies have shown that acute cocaine exposure produces widespread, cell-type-specific changes in gene expression across the fly brain (Baker et al., 2021b). Together, these findings indicate that stimulant related behaviors in flies are influenced by inherited genetic differences, and stimulant exposure itself induces transcriptional changes in the brain that may further contribute to these behaviors.

Human GWAS have also nominated candidate loci and genes associated with stimulant dependence and related traits (Gelernter et al., 2014; Cabana-Domínguez et al., 2019; Sherva et al., 2021), but such studies are not well-suited to uncover causal influences of candidate genes. Here again *Drosophila* are a great tool, as manipulation of the orthologs of these genes can determine whether a given association is causal or merely correlatory (Wangler et al., 2017). While human-GWAS-to-fly functional validation has yet to be applied systemically to SUD, it has been successfully used in alcohol research. In a GWAS of nearly 500,000 individuals from the UK, for example, 46 novel loci were associated with variation in daily alcohol consumption. Of these, the *hZip8* gene (alias: *SLC39A8*) was selected for further investigation due to its effect on putamen volume, which has itself been linked to alcohol consumption and withdrawal. Functional follow-up in flies revealed that overexpression of *hZip8* reduced naïve alcohol sensitivity, while knocking down the *hZip8* ortholog *dZip71B* increased naïve alcohol sensitivity and increased tolerance to a second exposure, suggesting a mechanistic link between the activity of this gene and the development of alcoholism (Evangelou et al., 2019). This example again highlights the mechanistic validity of *Drosophila* in studying stimulant use disorders and illustrates how fly genetics can complement human discovery-based approaches.

Flies are well-suited for genetic manipulation of dopamine signaling machinery. For instance, disrupting dDAT-dependent mechanisms alters amphetamine-related preference in flies (Belovich et al., 2021), supporting findings in mammals (Cagniard et al., 2014). Monoamine storage mechanisms also influence stimulant responses, as flies overexpressing dVMAT showed increased locomotor activity and grooming behaviors at baseline, but their sensitivity to cocaine was reduced. While VMAT/dVMAT are not primary targets of cocaine, this suggests that dVMAT modulation can induce molecular and neuronal adaptations that alter responses to stimulants (Chang et al., 2006).

At the anatomical level, studies have implicated both learning-related brain regions and sensory circuits in drug-related behaviors. Building on its established role in both sleep and dopamine-dependent learning (Lin, 2023), the mushroom body (MB) has also been linked to experience-dependent drug responses. For example, MB neurons have been implicated in experience-dependent alcohol-associated preference (Kaun et al., 2011), and other work has shown that disrupting MB development alters sensitivity to multiple drugs of abuse, including cocaine (King et al., 2011). Consistent with this, single-cell RNA sequencing after acute cocaine exposure identified prominent transcriptional responses in Kenyon cells of the MB, supporting the involvement of this brain region in cocaine-induced molecular responses (Baker et al., 2021b).

Together, these findings establish *Drosophila* as a versatile system for dissecting the biology of SUD. Flies link stimulant-related phenotypes to genetics, monoaminergic signaling, distinct brain regions, and sensory processing while also revealing how stimulant exposure changes gene expression in well-defined neuronal structures. Importantly, similar mechanisms shape sleep. This overlap positions *Drosophila* as a powerful model for studying sleep disturbances associated with SUD and identifying targets that may inform treatment of both.

### 3.5 *Drosophila* at the intersection of sleep and substance use disorders

Though studies on the specific interaction of sleep and SUD are sparse at time of writing, *Drosophila* have been used previously to identify drug-induced changes in sleep neurons in other substance use disorders. For example, although alcohol acutely functions as a sedative, it produces insomnia on subsequent nights, and this contributes to relapse in alcohol use disorder (Brower et al., 2011). Accordingly, a high dose of alcohol sedates flies, but after a night of recovery, sleep becomes shortened and fragmented for up to 8 days. Manipulation of different neurotransmitter systems using the thermogenetic activators and inhibitors described above revealed that this effect is mediated by alcohol-induced inhibition of distinct subsets of cholinergic neurons in the mushroom body (Chvilicek et al., 2025). Though not pertaining to stimulants specifically, studies like these demonstrate the power of flies in identifying specific drug-induced changes in neurons that regulate sleep.

The wake-promoting effects of caffeine have also been studied in flies. Caffeine is a non-amphetamine-type stimulant that primarily suppresses sleep by antagonizing adenosine receptors, which regulate sleep as described in Section 1. In flies, this adenosine effect was found to depend on caffeine’s ability to downregulate D1-type dopamine receptor expression in the MBs. This supports the critical role of dopamine in promoting drug-induced wakefulness but differs from amphetamine-type stimulants, which do not require modulation of MB dopamine receptors to exert their acute sleep effects (Andretic et al., 2008; Ferré, 2010). Notably, caffeine-induced sleep suppression is capable of prompting homeostatic rebound sleep (Brown et al., 2020; Segu and Kannan, 2023) and influencing circadian rhythms in flies; in the absence of external light cues, caffeine induces circadian phase delay (i.e., progressively increasing sleep latency, indicating a “daily” activity period of more than 24 h) at lower doses and arrhythmicity at higher doses, similar to the effect of METH in rodents. This is likely mediated by altered transcription of *timeless*, possibly facilitated by the known connections between DA neurons and clock neurons (Segu and Kannan, 2023).

Relatively few studies have directly investigated cocaine or amphetamine-type stimulant intake and sleep effects in flies, but existing work still provides valuable insight into this phenotype. Foundational studies demonstrated that methamphetamine feeding strongly suppresses sleep and can promote wakefulness even in sleep-deprived flies, supporting the face validity of fly stimulant responses (Andretic et al., 2005). This sleep suppression is dependent on D1-type receptor expression in brain areas outside the MB (Andretic et al., 2008). Work in flies has also demonstrated the necessity of DAT in mediating stimulant-induced sleep effects. As mentioned above, the *fumin* mutant was identified serendipitously as a low-sleeping mutant that was insensitive to sleep deprivation (Kume et al., 2005). Sleep in these flies is not suppressed by amphetamine feeding (Karam et al., 2022a), replicating earlier studies performed in *DAT* knockout mice (Giros et al., 1996; Wisor et al., 2001) and highlighting the mechanistic validity of the model. Paradoxically, feeding *dDAT*-null flies amphetamine or METH acutely enhances sleep to some extent, though the mechanism by which this occurs is unclear. While Gal-80^ts^-driven knockdowns enable temporal specificity in suppressing gene expression, the aforementioned lifelong *dDAT* knockouts leave open the possibility that compensatory mechanisms can develop during maturation (Karam et al., 2022a), making results from existing *DAT* knockout studies difficult to interpret. Finally, circadian systems also interact with stimulant responses, as both initial responses to cocaine and locomotor sensitization to the drug depend on proper function of circadian genes, especially *period* (Andretic et al., 1999; Filošević et al., 2018), and *Drosophila* dopamine receptor responsiveness fluctuates with circadian time (Andretic and Hirsh, 2000).

These foundational studies demonstrate the utility of flies in uncovering the basic genetic and molecular pathways linking sleep disorder and SUD. However, even though this work inspired many of the rodent studies described in Section 1, relatively few studies in flies have followed up on these findings, and there remain clear gaps in the literature. First, the effects of binge or chronic intermittent exposures in flies are relatively understudied compared to acute exposures. This is relevant because SUD often entails heavy or repetitive intake, which may induce different circuit adaptations than single brief exposures. Second, studies in flies rarely examine effects of drug abstinence, including both the initial withdrawal and long-term abstinence phases. As discussed in Section 1, the nature of sleep disturbance changes dynamically from early to late abstinence, so better understanding of the longitudinal nature of recovery from stimulant intake will be valuable for linking these changes to the neural mechanisms driving relapse. Finally, although sleep deprivation studies in *Drosophila* are fairly common, existing paradigms generally rely on acute sleep deprivation (e.g., 6 h of total sleep deprivation) rather than sleep deprivation of the type induced by stimulant use (i.e., several days of severely fragmented sleep), limiting the applicability of these findings to human SUD.

## 4 Conclusion

SUD is a complex condition with a substantial public health burden, yet treatment is difficult due to the lack of a pharmacological treatment option and a comparatively poor understanding of specific long-term stimulant-induced brain changes. Given the clinically relevant role of sleep disorder in driving relapse to SUD, there remains a need to dissect the genetic and neuromodulatory pathways driving sleep disorder during stimulant abstinence, as current research on the specific mechanisms linking SUD and sleep is lacking. The vinegar fly *Drosophila* boasts relevant sleep and stimulant responses, a substantial history of use identifying genes and neurotransmitter systems relevant to human pathology, and a wide variety of tools for dissecting neural systems relevant to SUD, making it a valuable model system for efficiently filling these gaps. Existing fly studies support a broadly wake-promoting role of dopamine, which can act at circadian, homeostatic, and action-selection circuits to suppress sleep and enable motivated behavior. Indeed, *Drosophila* replicate the initial stages of the human stimulant-sleep phenotype, with stimulants acutely suppressing sleep, dependent on dopamine signaling. Further research will likely identify specific changes in sleep circuits that occur over the course of stimulant use and abstinence to impair sleep and promote relapse.
